# Global prevalence and risk factors of *Enterocytozoon bieneusi* infection in humans: a systematic review and meta-analysis[Fn FN1]

**DOI:** 10.1051/parasite/2024007

**Published:** 2024-02-09

**Authors:** Yanchun Wang, Xiao-Man Li, Xing Yang, Xiang-Yu Wang, Yong-Jie Wei, Yanan Cai, Hong-Li Geng, Xin-Bo Yang, Hai-Long Yu, Hongwei Cao, Jing Jiang

**Affiliations:** 1 School of Pharmacy, Yancheng Teachers University Yancheng 224002 Jiangsu Province PR China; 2 College of Life Sciences, Changchun Sci-Tech University Shuangyang 130600 Jilin Province PR China; 3 Department of Technology, Ningbo Sansheng Biotechnology Co., Ltd Ningbo 315000 Zhejiang Province PR China; 4 College of Veterinary Medicine, Qingdao Agricultural University Qingdao 266109 Shandong Province PR China; 5 Department of Medical Microbiology and Immunology, School of Basic Medicine, Dali University Dali 671000 Yunnan Province PR China; 6 College of Animal Science and Technology, Jilin Agricultural University Changchun 130118 Jilin Province PR China

**Keywords:** *Enterocytozoon bieneusi*, Prevalence, Risk factors, Humans, Meta-analysis

## Abstract

*Enterocytozoon bieneusi* is one of the most important zoonotic pathogens. In this study, we present a systematic review and meta-analysis of the prevalence of human *E. bieneusi* infection in endemic regions and analyze the various potential risk factors. A total of 75 studies were included. Among 31,644 individuals tested, 2,291 (6.59%) were *E. bieneusi*-positive. The highest prevalence of *E. bieneusi* in the male population was 5.50%. The prevalence of *E. bieneusi* in different age groups was varied, with 10.97% in teenagers. The prevalence of *E. bieneusi* in asymptomatic patients (6.49%) is significantly lower than that in HIV-infected patients (11.49%), and in patients with diarrheal symptoms (16.45%). Rural areas had a higher rate (7.58%) than urban ones. The prevalence of *E. bieneusi* in humans was the highest (6.42%) at altitudes <10 m. Moreover, the temperate zone marine climate (13.55%) had the highest prevalence. A total of 69 genotypes of *E. bieneusi* have been found in humans. This is the first global study regarding *E. bieneusi* prevalence in humans. Not only people with low immunity (such as the elderly, children, people with HIV, etc.), but also people in Europe in temperate marine climates should exercise caution to prevent infection with *E. bieneusi* during contact process with animals.

## Abstract (fr)

*Enterocytozoon bieneusi* est l’un des agents pathogènes zoonotiques les plus importants. Dans cette étude, nous présentons une revue systématique et une méta-analyse de la prévalence de l’infection humaine à *E. bieneusi* dans les régions endémiques et analysons les différents facteurs de risque potentiels. Au total, 75 études ont été incluses. Parmi 31 644 individus, 2 291 (6,59 %) étaient positifs à *E. bieneusi*. La prévalence la plus élevée d’*E. bieneusi* dans la population masculine était de 5,50 %. La prévalence d’*E. bieneusi* dans différents groupes d’âge variait, avec 10,97 % chez les adolescents. La prévalence d’*E. bieneusi* chez les patients asymptomatiques (6,49 %) était significativement inférieure à celle des patients VIH (11,49 %) et des patients présentant des symptômes de diarrhée (16,45 %). Les zones rurales avaient un taux plus élevé (7,58 %) que les zones urbaines. La prévalence d’*E. bieneusi* chez les humains était la plus élevée (6,42 %) à une altitude <10 m. De plus, le climat marin de la zone tempérée (13,55 %) avait la prévalence la plus élevée. Au total, 69 génotypes d’*E. bieneusi* ont été trouvés chez l’homme. Il s’agit de la première étude mondiale concernant la prévalence d’*E. bieneusi* chez l’homme. Non seulement les personnes ayant une faible immunité (telles que les personnes âgées, les enfants, les patients atteints du VIH, etc.), mais également les personnes vivant en Europe dans un climat marin tempéré doivent veiller à prévenir l’infection par *E. bieneusi* lors du contact avec des animaux.

## Introduction

The Microsporidia, infecting a broad range of both vertebrate and invertebrate hosts, live exclusively within host cells [30]. This group of parasites currently comprises more than 200 genera and 1,600 species, including relatively common species, such as *Encephalitozoon cuniculi, Enterocytozoon bieneusi, Encephalitozoon intestinalis*, and *Encephalitozoon hellem* [5, 75]. *Enterocytozoon bieneusi* is the most important zoonotic microsporidian species worldwide and is transmitted predominantly through the fecal-oral route because it is capable of infecting a broad range of hosts, including humans, domestic animals, poultry, companion animals, birds, and wildlife [14, 63].

*Enterocytozoon bieneusi* was first identified in human immunodeficiency virus (HIV) patients in 1985, and with the expansion of the HIV epidemic, the number of cases of *E. bieneusi* infections in humans has increased [29]. To date, more than 200 genotypes have been identified and classified into 11 groups (groups 1–11) [40]. The genotypes identified in Group 1 are predominantly present in the human population, with the most frequently occurring ones being A, D, EbpC, and Type IV [40]. Although genotypes from other groups can also infect humans, they are relatively rare and are generally considered to have little public health significance [42].

*Enterocytozoon bieneusi* infection is associated with persistent diarrhea, malabsorption, and wasting diathesis in individuals with compromised immune systems, particularly those diagnosed with acquired immunodeficiency syndrome (AIDS) and can lead to life-threatening chronic diarrhea [6, 51, 87]. Patients with normal immune function can also develop self-limited diarrhea lasting up to one month [70]. Unfortunately, there are no currently effective treatments available for *E. bieneusi* infection [41]. Due to its importance and potential threat to public health, *E. bieneusi* has been classified as a Category B agent by the National Institutes of Health (NIH, https://www.niaid.nih.gov/research/emerging-infectious-diseases-pathogen), making it a second-highest priority organism/biological agent [97]. In addition, many public health organizations and academic institutions in various countries and regions have included *E. bieneusi* in their monitoring and control plans for infectious pathogens to ensure public health and safety.

Here, we present a systematic review and meta-analysis that evaluates the prevalence of human *E. bieneusi* infection in endemic regions. Our analysis considered various potential risk factors, such as gender, age, season, and geographic location.

## Material and methods

### Search strategy

We searched for all studies on the prevalence of *E. bieneusi* infection in humans around the world up to July 2022 from six databases, *i.e*., China National Knowledge Infrastructure (CNKI), VIP Chinese Journal Database (VIP), Wanfang Data, PubMed, Web of Science, and ScienceDirect. The retrieval strategy for the three Chinese databases was to use the search keywords “humans (in Chinese)” and “Microsporidia (in Chinese)”, in advanced retrieval. The three English-language databases were searched using search formulas, as follows: **Web of Science**: (TI=(Humans) OR TI=(Man) OR TI=(Homo sapiens)) AND (TI=(Microsporidia) OR TI=(Microsporidium) OR TI=(Microsporidiums)); **ScienceDirect**: (Human OR “Homo sapiens” OR Man) AND (Microsporidia OR Microsporidiums OR Microsporidium); **PubMed**: ((“Humans”[Mesh]) OR (Homo sapiens[Title/Abstract]) OR (Man[Title/Abstract]) OR (Man, Modern[Title/Abstract]) OR (Modern Man[Title/Abstract]) OR (Human[Title/Abstract])) AND (“Microsporidia”[Mesh]) OR (Microsporidias[Title/Abstract]) OR (Microspora[Title/Abstract]) OR (Microsporidians[Title/Abstract]) OR (Microsporidian[Title/Abstract]) AND (“epidemiology” [MeSH]) OR (epidemics[Title/Abstract])) OR (prevalence[Title/Abstract])) OR (frequency[Title/Abstract])) OR (surveillance[Title/Abstract])) OR (incidence[Title/Abstract])) OR (occurrence[Title/Abstract])).

### Inclusion and exclusion criteria

The literature aggregation software used was Endnote X9.3.2 [15]. The inclusion criteria for this meta-analysis were as follows: (1) study on the prevalence of *E. bieneusi* in humans; (2) data must include a clear total number of surveyed humans and the number of positives; (3) the articles must have full text; and (4) the research must be designed to scale out. The exclusion criteria for this meta-analysis were as follows: (1) not Chinese or English literature; (2) data error, no data or data duplication in studies; (3) the research subject is not *E. bieneusi* and humans; (4) conference report or summary; and (5) no detailed positivity rate.

### Data extraction

Two authors independently extracted and recorded the data. The lead author of this meta-analysis further evaluated any differences or uncertainties regarding research qualifications. The extracted data included the article title, testing method, residence, HIV or diarrhea, article quality, age and gender of the patient, country, longitude and latitude, altitude and climate, and the total and positive numbers. The geographic data (longitude, latitude, and altitude) collected were from the National Oceanic and Atmospheric Administration (NOAA; https://www.ncei.noaa.gov/maps/monthly/). We also considered various socioeconomic variables (World Bank–income category, National population in 2021, and the human development index (HDI): https://data.worldbank.org/, https://population.un.org/, and https://hdr.undp.org/).

### Statistical analyses

This meta-analysis was performed according to the PRISMA statement [67]. The “meta” package (version 6.0-0) in RStudio (version 4.0.5) was employed to analyze the data. Before performing the meta-analysis, we tested five transformation methods (Table S1). The *W*-value close to 1 and the *p-*value > 0.05 were close to the normal distribution criterion. We used Cochran-*Q*, *I*^*2*^ statistics, and *χ*^2^ tests to calculate the heterogeneity between studies. When *p*-value < 0.05 and *I*^2^ > 50%, this indicates the existence of heterogeneity, and the random effect model was adopted; when *p*-value > 0.05, *I*^*2*^ < 50%, this indicates that there is no heterogeneity, and the fixed effect model was adopted. A forest plot was used to visualize the statistical results of the meta-analysis; a funnel plot and Egger test detected the publication bias of the research; the sensitivity analysis evaluated the stability of the meta-analysis model and the reliability of the results; and subgroup analysis and univariate regression analysis verified the potential source of heterogeneity.

## Results

### Search results

**Figure 1:**
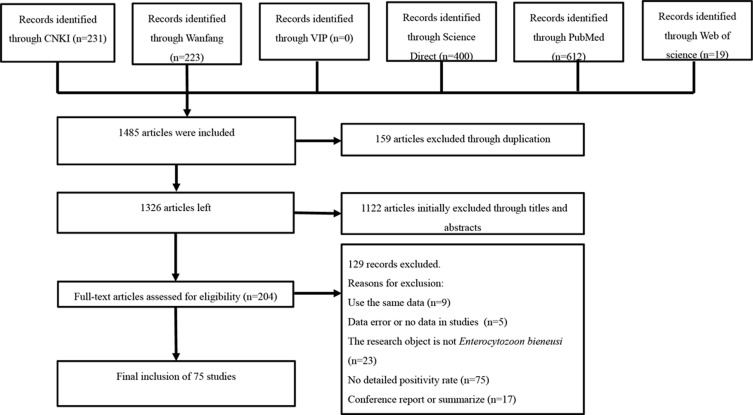
Flow diagram of literature search and selection.

A total of 1,485 publications were identified, and after the titles and abstracts were reviewed, 1,326 papers were selected for full-text reading. According to the inclusion and exclusion criteria, 75 studies were finally included after screening (Fig. 1 and Table S2). Among them, 40 publications had 4 points, 26 publications had 3 points, and 9 publications had 2 points (Table S2).

### Qualification research and publication bias

**Figure 2:**
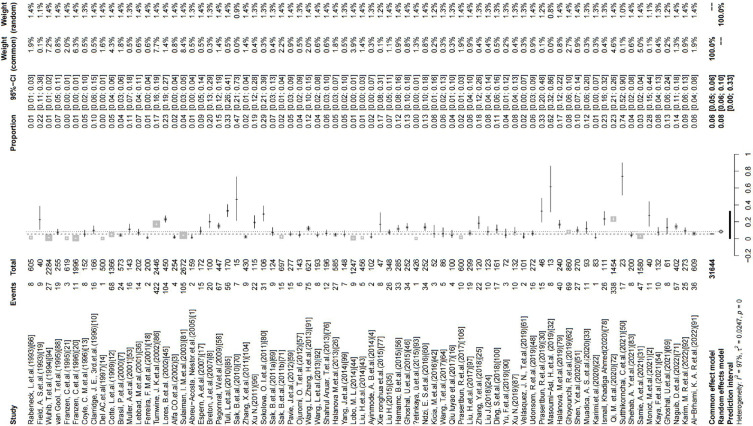
Forest map of global human infection in *E. bieneusi*.

**Figure 3:**
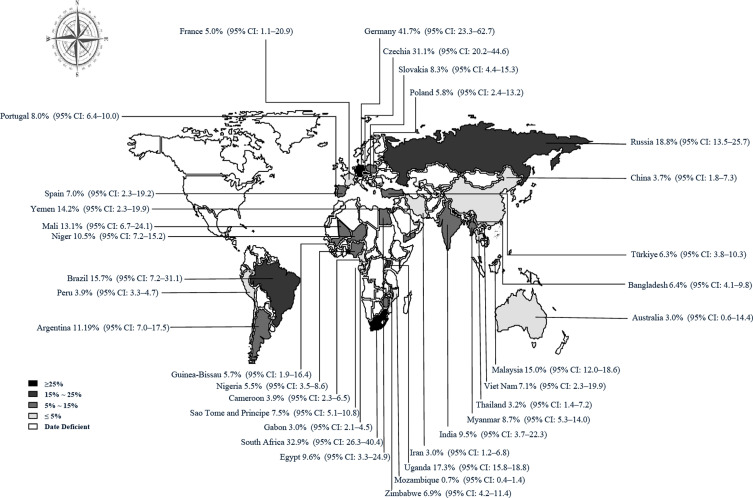
Map of *E. bieneusi* prevalence in human worldwide.

This study used PLOGIT for data transformation (Table S1; *W* = 0.98608, *p* = 0.5848). The forest plot of global human infection with *E. bieneusi* showed high heterogeneity (*χ*^2^ = 1.6138, *I*^2^ = 96.0%, *p* < 0.01), so a random effects model was used (Fig. 2). According to the funnel plot and Egger test, there was publication bias in this study (*p* = 0.0001; Table S3, Figs. S1, and S2). There were 24 supplementary articles shown in the trim and fill analysis (Fig. S3). Based on the sensitivity test, the data after restructuring were not significantly affected (Fig. S4). Figure 3 shows the distribution of human *E. bieneusi* prevalence. Distribution is essentially worldwide, but there are few reports from North America.

### Meta-analysis of *E. bieneusi* prevalence in humans worldwide

Detailed data on global human *E. bieneusi* prevalence are summarized in Tables 1 and 2. The global prevalence of *E. bieneusi* infection in humans was 6.59% (95% CI: 4.97–8.68). The highest prevalence of *E. bieneusi* in the male population was 5.50% (95% CI: 3.54–8.45). Concerning the environment of residence, rural areas had the highest rate at 7.58% (95% CI: 4.22–13.25). The prevalence of *E. bieneusi* in teenagers was 10.97% (95% CI: 5.90–17.36). The infection rate was 16.5% (95% CI: 11.47–22.18) in patients with diarrhea, and 10.54% (95% CI: 5.79–16.44) in patients without diarrhea. The rate of infection in patients with cancer was 69.89% (95% CI: 60.13–78.84), and the rate of infection in other patients was also high, *i.e*., 39.00% (95% CI: 32.34–45.87) in bone marrow transplant patients, 14.4% (95% CI: 9.84–19.62) in patients with HIV, etc. Concerning the analyzed geographic factors, the positive rate of *E. bieneusi* was the highest in humans living at altitudes <10 m (6.42%; 95% CI: 4.02–10.08). According to the analysis of climatic factors, we found that the temperate zone marine climate had the highest positive rate of human *E. bieneusi* (13.55%; 95% CI: 3.19–42.73; Table 1).

**Table 1:** Summary of global *E. bieneusi* human infection rates and relevant characteristics.

Variable/Category	No. studies	No. examined	No. positive	Prevalence % (95% CI)	Heterogeneity			Univariate meta-regression	
					*χ* ^2^	*p-*value	*I*^2^ (%)	*p-*value*	Coefficient (95% CI)
Gender								0.6839	0.1505 (−0.5737 to 0.8746)
Female	31	6,507	389	4.66% (2.68–7.98)	449.44	<0.01	93.3		
Male	32	8,235	497	5.50% (3.54–8.45)	477.02	<0.01	93.5		
Residence								0.8192	0.1173 (−0.8888 to 1.1234)
Urban	4	717	55	6.98% (3.80–12.49)	13.34	<0.01	77.5		
Rural	8	2,232	166	7.58% (4.22–13.25)	88.83	<0.01	92.1		
Age								0.3148	−0.0508 (−0.2047 to 0.1030)
Teenager	26	8,190	912	10.97% (5.90–17.36)	244.58	<0.01	97.2		
Adult	22	9,243	239	3.91% (2.32–5.88)	886.42	<0.01	91.4		
Elderly	7	816	75	6.75% (2.95–11.94)	33.12	<0.01	81.9		
Human Development Index								0.1191	0.5251 (−0.1353 to 1.1855)
Very high	28	8,120	974	7.93% (4.94–12.48)	544.06	<0.01	95.0		
High	27	15,257	613	4.84% (2.96–7.83)	542.24	<0.01	95.2		
Medium	10	2,918	252	7.32% (4.73–11.14)	145.64	<0.01	93.8		
Low	8	4,761	541	6.87% (3.45–13.22)	128.25	<0.01	94.5		
Income level								0.0638	−0.6883 (−1.4161 to 0.0396)
High	18	5,099	736	9.53% (5.01–17.37)	357.78	<0.01	95.2		
Upper-middle	31	16,717	779	5.07% (3.24–7.85)	636.82	<0.01	95.3		
Lower-middle	15	3,970	320	7.31% (5.17–10.24)	184.31	<0.01	92.4		
Low	5	4,034	466	6.36% (2.23–16.82)	106.73	<0.01	96.3		
Clinical signs and symptoms								0.009	0.0646 (0.1612 to −01.1311)
AIDS	1	2,53	28	11.07% (7.47–15.2)	0.00	–	–		
HIV	27	7,920	869	14.4% (9.84–19.62)	833.11	<0.01	96.9		
Leukemia	3	280	33	10.52% (1.83–24.53)	20.90	<0.01	90.4		
Malignancies	1	53	3	5.6% (0.73–13.85)	0.00	–	–		
Cancer	1	93	65	69.89% (60.13–78.84)	0.00	–	–		
Bone marrow transplant	1	200	78	39.00% (32.34–45.87)	0.00	–	–		
CD4^+^								0.7435	−0.0535 (−0.3736 to 0.2667)
<200	8	921	103	25.97% (9.74–46.19)	230.94	<0.01	97.0		
>200	6	554	81	21.76% (4.66–46.00)	69.73	<0.01	92.8		
Diarrhea								0.0948	0.0928 (−0.0161 to 0.2016)
Yes	31	4,660	763	16.50% (11.47–22.18)	344.45	<0.01	91.3		
No	20	7,919	578	10.54% (5.79–16.44)	523.67	<0.01	96.4		
Altitude (m)								0.4657	0.2374 (−0.4004 to 0.8753)
<10 m	28	7,559	591	6.42% (4.02–10.08)	341.40	<0.01	92.1		
10 m–50 m	21	9,611	339	4.68% (2.91–7.44)	292.93	<0.01	93.2		
>50 m	14	6,032	317	5.96% (2.97–11.61)	289.20	<0.01	95.5		
Climate								0.1625	0.9607 (−0.3875 to 2.3089)
Mediterranean	10	1,741	119	5.74% (2.99–10.76)	67.98	<0.01	86.8		
Savannah	6	1,381	117	10.26% (4.59–21.36)	131.12	<0.01	96.2		
Tropical monsoon	13	3,539	272	4.54% (2.32–8.68)	174.38	<0.01	93.1		
Tropical desert	5	4,160	238	7.50% (4.77–11.60)	77.49	<0.01	94.8		
Tropical rainforest	4	1,712	123	7.03% (3.75–12.79)	55.30	<0.01	94.6		
Temperate continental	8	3,518	114	5.01% (1.54–15.08)	183.55	<0.01	96.2		
Temperate zone marine climate	5	2,112	372	13.55% (3.19–42.73)	85.24	<0.01	95.3		
Temperate monsoon	5	3,945	123	5.23% (1.98–13.09)	90.65	<0.01	95.6		
Subtropical monsoon	11	2,959	208	5.95% (2.84–12.03)	72.22	<0.01	86.2		
Total	75	31,644	2291	6.59% (4.97–8.68)					

CI: confidence interval; NA: not applicable.

* *p* < 0.05 is statistically significant.

**Table 2:** Prevalence estimates of *E. bieneusi* infection, and estimated numbers of infected people in 34 countries.

Country and continent (No.)	No. examined	No. positive	% (95% CI)	Estimated population size 2021 (thousands)	Estimated number of infected people (95% CI)
Australia (3)	845	48	2.97% (0.56–14.35)	25,921	770 (145–3,720)
**Oceania**	845	48	4.52% (0.01–16.52)	44,492	2,011 (4–7,350)
Argentina (1)	143	16	11.19% (6.97–17.48)	45,277	5,066 (3,156–7,914)
Brazil (2)	206	27	15.73% (7.16–31.13)	214,326	33,713 (15,346–66,720)
Peru (1)	2672	105	3.93% (3.26–4.74)	33,715	1,325 (1,099–1,598)
**South America**	3021	148	10.99% (4.12–20.63)	434,254	47,724 (17,891–89,586)
China (16)	9826	328	3.66% (1.81–7.25)	1,425,893	52,188 (25,809–103,377)
Malaysia (1)	447	67	14.99% (11.97–18.61)	33,574	5,033 (4,019–6,248)
Myanmar (1)	172	15	8.72% (5.33–13.96)	53,798	4,691 (2,867–7,510)
Thailand (5)	2049	111	3.18% (1.37–7.22)	71,601	2,277 (981–5,170)
**East and South-East Asia**	12494	521	5.22% (3.19–7.71)	2,339,493	122,121 (74,629–180,374)
Bangladesh (1)	299	19	6.35% (4.09–9.75)	169,356	10,754 (6,927–16,512)
India (4)	943	144	9.48% (3.68–22.31)	1,407,564	133,437 (51,798–314,028)
**Central and South Asia**	1242	163	4.67% (3.08–7.01)	2,065,350	96,451 (63,612–144,781)
Iran (3)	834	22	2.93% (1.24–6.80)	87,923	2,576 (1,090–5,979)
Türkiye (2)	223	14	6.28% (3.75–10.32)	84,775	5,324 (3,179–8,749)
Yemen (1)	402	57	14.18 (11.10–17.94)	32,982	4,677 (3,661–5,917)
**North Africa and Western Asia**	1459	93	5.60% (2.39–10.04)	545,471	30,546 (13,036–54,765)
Cameroon (2)	1018	37	3.88% (2.28–6.53)	27,199	1,055 (620–1,776)
Egypt (2)	685	47	9.62% (3.30–24.93)	109,262	10,511 (3,606–27,239)
Gabon (1)	758	22	3.04% (2.06–4.46)	2,341	71 (48–104)
Guinea-Bissau (1)	52	3	5.77% (1.87–16.42)	2,061	119 (39–338)
Mali (1)	61	8	13.11% (6.70–24.10)	21,905	2,872 (1,468–5,279)
Mozambique (1)	1247	9	0.72% (0.38–1.38)	32,077	231 (122–443)
Niger (1)	228	24	10.53% (7.16–15.22)	25,253	2,659 (1,808–3,844)
Nigeria (2)	325	18	5.54% (3.52–8.62)	213,401	11,822 (7,512–18,395)
South Africa (1)	170	56	32.94% (26.30–40.35)	59,392	19,564 (15,620–23,965)
Sao Tome and Principe (1)	348	26	7.47% (5.14–10.75)	NA	NA
Uganda (1)	2446	422	17.25% (15.81–18.80)	45,854	7,910 (7,250–8,621)
Vietnam (1)	42	3	7.14% (2.32–19.93)	97,468	6,959 (2,261–19,425)
Zimbabwe (1)	202	14	6.93% (4.15–11.36)	15,994	1,108 (664–1,817)
**Africa**	7582	689	8.06% (4.91–11.92)	1,137,939	91,717 (55,872–135,642)
Czechia (3)	560	188	31.08% (20.15–44.63)	10,511	3,267 (2,118–4,691)
France (3)	599	11	5.04% (1.05–20.90)	64,531	3252 (678–13,487)
Germany (2)	59	23	41.66% (23.28–62.70)	83,409	34,748 (19,418–52,297)
Poland (1)	86	5	5.81% (2.44–13.22)	38,308	2,226 (935–5,064)
Portugal (1)	860	69	8.02% (6.39–10.04)	10,290	825 (658–1,033)
Russia (1)	159	30	18.87% (13.52–25.71)	145,103	27,381 (19,618–37,306)
Slovakia (2)	233	20	8.33% (4.37–15.30)	5,448	454 (238–834)
Spain (3)	403	34	6.96% (2.31–19.16)	47,487	3,305 (1,097–9,099)
**Europe**	4,413	718	14.70% (8.21–22.68)	745,174	109,540 (61,178–169,005)
**World**	31,644	2291	6.59% (4.97–8.68)	7,909,295	521,222 (393,092–686,526)

CI: confidence interval; NA: not applicable.

According to income level, the highest prevalence rates of *E. bieneusi* infection was in countries with high (9.53%; 95% CI: 5.01–17.37) and lower-middle (7.31%; 95% CI: 5.17–10.24) income levels, with the lowest prevalence estimated for upper-middle income countries (5.07%; 95% CI: 3.24–7.85). According to HDI, the level subgroup analysis indicated that the highest prevalence rates were estimated for countries with extremely high HDI levels (7.93%; 95% CI: 4.94–12.48%) (Table 2). Countries with high prevalence rates included Germany (41.66%), South Africa (32.94%), Czechia (31.08%), and Russia (18.87%). The highest positive rate of human *E. bieneusi* infection was 14.70% (95% CI: 8.21–22.68) in Europe, which indicates that 109,540,000 (range: 61,178,000–169,005,000) people will be infected in Europe in 2021. An extrapolation to the 2021 world population indicated that 521,222,000 (range: 393,092,000–686,526,000) people harbored *E. bieneusi* infection. More detail on the global and regional *E. bieneusi* infection prevalence is given in Table 2.

**Figure 4:**
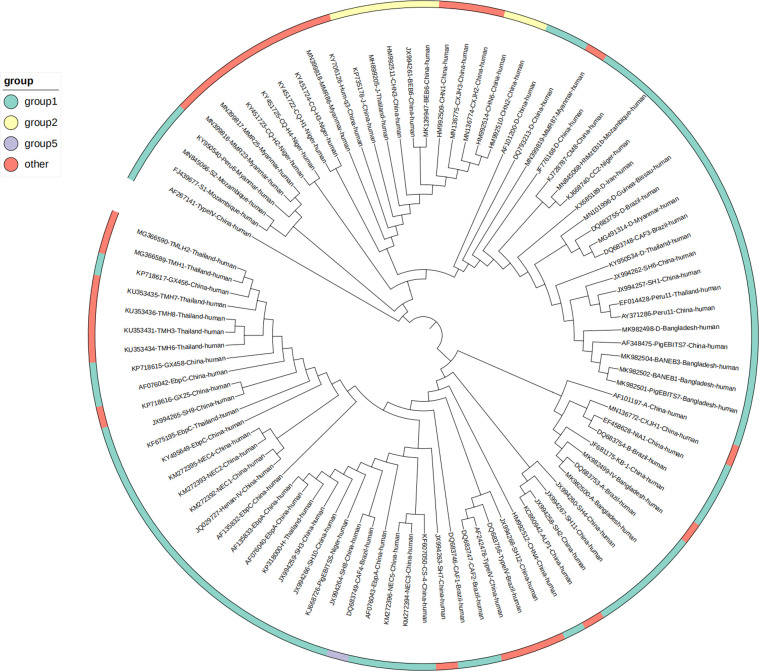
Evolutionary tree of *E. bieneusi* prevalence in human worldwide.

**Figure 5:**
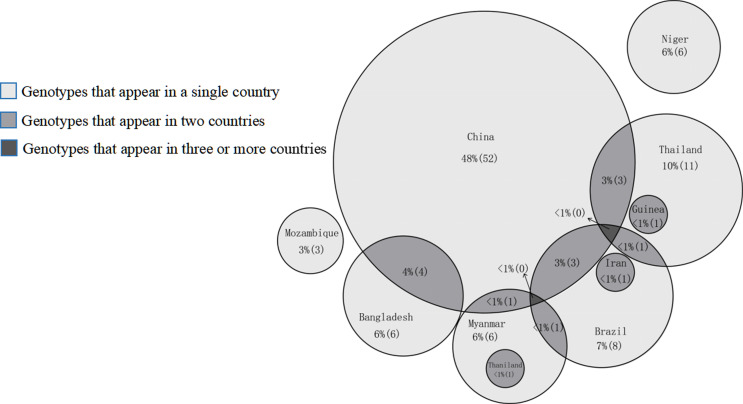
Prevalence of *E. bieneusi* genotype in different countries.

A total of 70 *E. bieneusi* genotypes were included in this study, of which Group 1 was the most common in human infections (*n* = 40, 57.14%), followed by Group 2 (*n* = 5, 7.14%) (Table 3 and Fig. 4). According to the genotypes of *E. bieneusi* in different countries, China had the most *E. bieneusi* genotypes (*n* = 52; 48%). China shares three genotypes with Brazil and Thailand, four with Bangladesh, and one with Myanmar (Fig. 5).

**Table 3:** Prevalence of *E. bieneusi* genotype grouping.

Group	Number of species detected/prevalence (%)	Number of positives detected/prevalence (%)
Group 1	40 (57.14)	266 (82.05)
Group 2	5 (7.14)	27 (7.69)
Group 5	1 (1.42)	9 (2.56)
Other	24 (34.28)	57 (16.24)
Total	70 (100)	351 (100)

**Group 1**: D (102; 29.06%), EbpC (51; 14.53%), Type IV/K (30; 8.55%), NIA1 (19; 5.41%), A/Ind 4 (18; 5.13%), Peru6 (10; 2.85%), Peru11 (6; 1.71%), EbpA (5; 1.42%), HhMzEb1b (4; 1.14%), B (3; 0.85%), CAF1 (3; 0.85%), CHN4 (3; 0.85%), Henan-IV (3; 0.85%), SH2 (3; 0.85%), CC2 (2; 0.57%), CS-4 (2; 0.57%), BANEB1 (1; 0.28%), BANEB3 (1; 0.28%), CAF2 (1; 0.28%), CAF3 (1; 0.28%), CM8 (1; 0.28%), GX458 (1; 0.28%), H (1; 0.28%), KB-1 (1; 0.28%), NEC2 (1; 0.28%), NEC3 (1; 0.28%), NEC4 (1; 0.28%), NEC5 (1; 0.28%), Peru12 (1; 0.28%), S1 (1; 0.28%), S2 (1; 0.28%), SH1 (1; 0.28%), SH10 (1; 0.28%), SH11 (1; 0.28%), SH3 (1; 0.28%), SH4 (1; 0.28%), SH6 (1; 0.28%), SH8 (1; 0.28%), SH9 (1; 0.28%), GX456 (1; 0.28%).

**Group 2**: CHN3 (4; 1.14%), J (3; 0.85%), CHN2 (2, 0.57%), SH5/BEB6 (2; 0.57%), CHN6 (2; 0.28%).

**Group 5**: CAF4 (9; 2.56%).

**Other**: CHN1 (5; 1.42%), TMH6 (4; 1.14%), ALP1 (3; 0.85%), TMH3 (3; 0.85%), TMH7 (3; 0.85%), TMLH2 (3; 0.85%), CQ-H2 (2; 0.57%), CQ-H3 (2; 0.57%), TMH1 (2; 0.57%), CQ-H1 (1; 0.28%), CQ-H4 (1; 0.28%), CXJH1 (1; 0.28%), CXJH2 (1; 0.28%), CXJH3 (1; 0.28%), F (1; 0.28%), GX25 (1; 0.28%), MMR23 (1; 0.28%), MMR25 (1; 0.28%), MMR86 (1; 0.28%), MMR87 (1; 0.28%), SH12 (1; 0.28%), SH7 (1; 0.28%), TMH2 (1; 0.28%), TMH8 (1; 0.28%).

## Discussion

Microsporidia specializing in intracellular parasitism [5] are a type of single-celled eukaryotic organism [29, 76]. As we know, more than 90% of human microsporidiosis cases are caused by *E. bieneusi* as the most important species of microsporidiosis, with worldwide distribution of infected cases [37, 65, 83, 94]. We have estimated that of the world population of 7.9 billion people, over 520 million people may be infected with *E. bieneusi* in 2021 [10].

In this study, the results showed that the infection rate in men was higher than that in women, but the difference was not statistically significant. A similar phenomenon was also found in the meta-analysis of the prevalence of microsporidia in China by Qiu *et al*. [65]. For people, the high male infection rate may be related to poor living habits and higher engagement in animal husbandry. At the same time, the high positive rate of the population in rural areas may also be related to high engagement in animal husbandry [55]. In rural areas, it is customary to raise poultry, livestock and pets, and most of them are raised in a way that combines free range breeding with captive breeding. Close contact between people and animals, poor living conditions, pollution of drinking water by animal feces, and low awareness of prevention methods all increase the probability of *E. bieneusi* infection [41].

There are currently more than 500 genotypes of *E. bieneusi* identified based on ITS nucleic acid sequences, and these existing genotypes can be divided into different genetic groups, totaling 11 groups (Groups 1–11). The results of this study showed that 82.05% of genotypes belonged to Group 1. Group 1 has the largest number of species and is found in both domestic and wild animals worldwide [42], and is also the genotype group of *E. bieneusi* that mainly infects humans [31, 32]. However, genotypes in other groups causing zoonotic disease cannot be ignored, such as J and BEBE4 in group 2, CAF4 and KIM3 in group 5, Nig3 and Nig4 in group 6, and S7 in group 10 [31, 40, 53, 63]. These genotypes with the potential for zoonotic co-infection, can infect both animal hosts and humans, establishing a pathway of transmission between humans and animals [41, 104, 105]. This is therefore of high public health significance. Findings also further demonstrate that human *E. bieneusi* infection is mainly related to animals [31, 32].

*Enterocytozoon bieneusi* is one of the common pathogens that causes chronic diarrhea in the human body, especially in people with immunodeficiency or low immunity (such as people with HIV, organ transplant recipients, bone marrow transplant recipients, patients with tumors, elderly people, and children) [83, 94, 100]. In the statistical analysis of people at different ages, compared with other groups, the infection rates of young people and the elderly were higher. Previous studies have shown that young and older age are risk factors for *E. bieneusi* infection [29]. The prevalence of *E. bieneusi* in diarrhea patients 16.50% (11.47–22.18) was significantly higher than that in non-diarrhea patients 10.54% (5.79–16.44). Several studies have shown similar results, indicating that patients with diarrhea have significantly higher rates of *E. bieneusi* infection than those without diarrhea. People with diarrhea are more susceptible to infection [99, 102]*.* Meanwhile, *E. bieneusi* infection is widespread and infectious for people with HIV, as well as for others with compromised immunity [29]. This study found that *E. bieneusi* had the highest infection rate among patients with cancer, followed by organ transplant recipients. However, the mechanism of infection and pathogenicity established by the entry of *E. bieneusi* into host cells is not yet clear, and there may be a certain balance between it and the host, causing the host to be in a subclinical state for a long period of time [74]. Once the host’s immune function is compromised, obvious clinical symptoms will appear, such as persons with ≤200 CD4^+^ T cells per microliter blood [16, 26]. Moreover, the infection rate of *E. bieneusi* in persons with <200 (25.97%) CD4^+^ T cells was higher than that in those with >200 CD4^+^ T cells (21.76%). Therefore, it is recommended that patients with low immunity should maintain good hygiene habits and seek medical attention in good time when symptoms such as diarrhea occur [101, 106].

Our analysis also showed that based on economic development, high infection rates are found in the extremely high HDI and high-income countries. On the one hand, this may be due to differences in testing technology, focus, and logistics development [69]. On the other, it may be due to climate. Our research showed that the prevalence rate of *E. bieneusi* in European countries was relatively high. The results of the climate subgroups showed that the prevalence in temperate zone marine climate was the highest. Studies have shown that extreme temperatures and precipitation are not conducive to the growth of *E. bieneusi* [43, 63]. The temperate zone marine climate has the characteristics of warm winters and cool summers, small annual temperature differences, and uniform precipitation distribution. This may be beneficial for the growth and transmission of *E. bieneusi*.

Although we conducted a comprehensive and detailed analysis of the risk factors for *E. bieneusi* infection in humans in this study, there were certain limitations. First, we may have missed some studies. Second, in some cases, our estimates might not be representative of national prevalence nor of all communities in a country. Third, some included studies lack infection rates based on gender, age, and living environment factors, and information on the different detection methods used, which may affect certain subgroup analyses.

## Conclusions

This study revealed a global prevalence rate of 6.59% for human *E. bieneusi* infection, with 82.05% of the genotypes belonging to Group 1 and posing a risk of zoonotic disease. Not only people with low immunity (such as the elderly, children, patients with HIV, *etc*.), but also people in Europe living in temperate marine climates should exercise caution to prevent infection with *E. bieneusi* during contact with animals.

## Supplementary material

The supplementary material of this article is available at https://www.parasite-journal.org/10.1051/parasite/2024007/olm.*Table S1*: Normal distribution tests for normal rates and different transitions of articles hosted by humans.*Table S2*: Main characteristics of the included studies in humans.*Table S3*: Egger for publication bias.**Figure S1:**
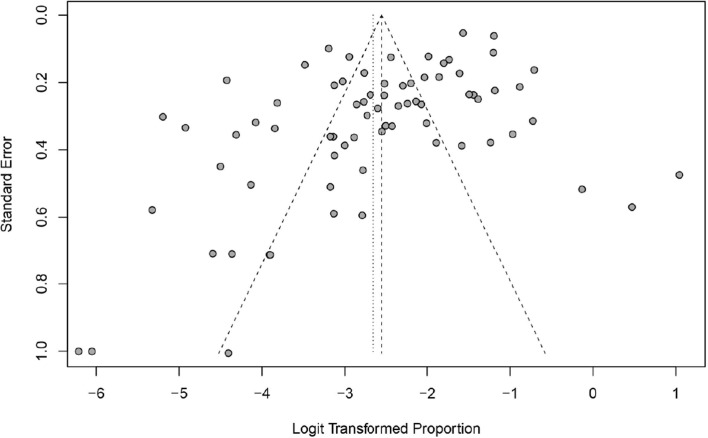
Funnel plot with pseudo 95% confidence interval for publication bias test.**Figure S2:**
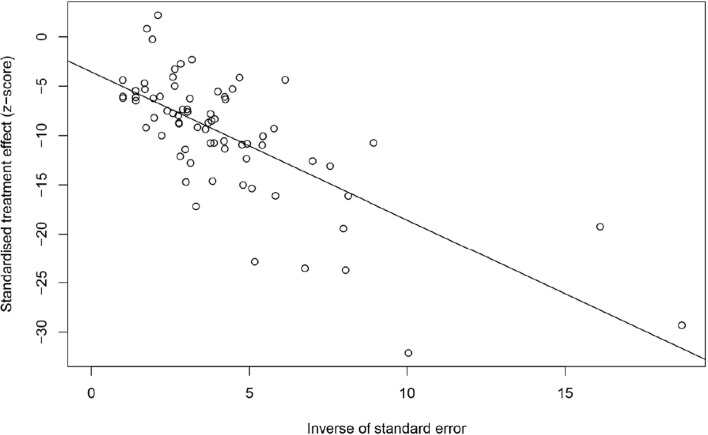
Egger’s test for publication bias.**Figure S3:**
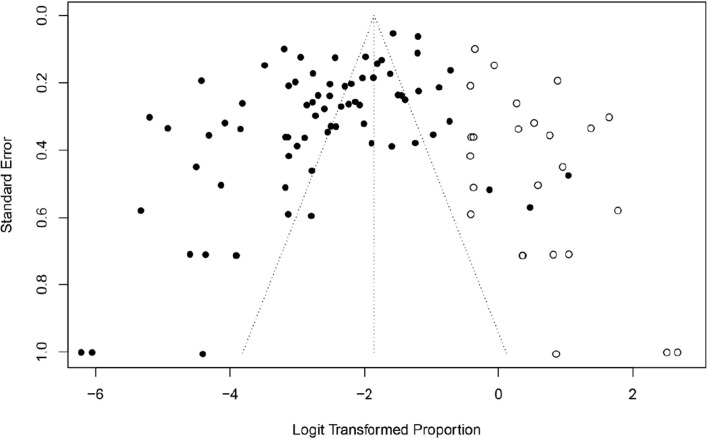
Shear complement graph and pseudo 95% confidence interval for publication bias test.**Figure S4:**
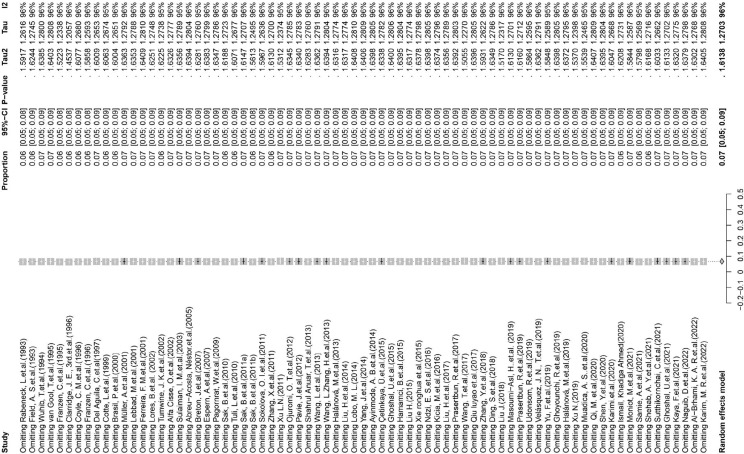
*Figure S4*: Sensitivity analysis of human infection with *E. bieneusi*.

## Data Availability

The data used to support the findings of this study are available from the corresponding authors upon request.
