# Supplementary material for: Global prevalence and risk factors of Enterocytozoon bieneusi infection in humans: a systematic review and meta-analysis
Source: Parasite. 2024 Feb 9;31:9. doi: 10.1051/parasite/2024007 (PMC10860563; doi:10.1051/parasite/2024007)
Supplement: Supplementary file 1 — Table S1: Normal distribution tests for normal rates and different transitions of articles hosted by humans. [file parasite-31-9-s1.pdf]

Table S1 Normal distribution tests for normal rates and different transitions of articles hosted by humans.

**Conversion form**	**W**	**P**
PRAW	0.7187	1.091e-10
PLN	0.9718299	0.09258
PLOGIT	0.98608	0.5848
PAS	0.90758	4.466e-05
PFT	0.86679	1.202e-06

"PRAW": raw exchange rate; PLN: log conversion. "PLOGIT": logit transformation;

"PAS": arcsine transformation; "PFT": double arcsine transformation;
