# Supplementary material for: Global prevalence and risk factors of Enterocytozoon bieneusi infection in humans: a systematic review and meta-analysis
Source: Parasite. 2024 Feb 9;31:9. doi: 10.1051/parasite/2024007 (PMC10860563; doi:10.1051/parasite/2024007)
Supplement: Supplementary file 2 — Table S2: Main characteristics of the included studies in humans. [file parasite-31-9-s2.pdf]

Table S2. Main characteristics of the included studies in humans.

First author's last name/ Pub year (Ref)	Country	Diagnostic Method	Human Development [illegible]	Level of Income	Sample size	Infected Total	Score
**Rabeneck, L.et.al.(1993) [66]**	NA	Microscopic examination	NA	NA	106	31	3
**Field, A. S.et.al.(1993) [19]**	Australia	Microscopic examination	Very high	High	240	40	5
**Wuhib, T.et.al.(1994) [94]**	Brazil	PCR	High	Upper-middle	72	3	4
**van Gool, T.et.al.(1995) [88]**	Zimbabwe	Microscopic examination	Medium	Upper-middle	202	14	5
**Franzen, C.et.al.(1995) [21]**	Germany	PCR	Very high	Lower-middle	13	8	3
**Franzen, C.et.al.(1996) [20]**	Cameroon	PCR	Medium	High	23	17	3
**Coyle, C. M.et.al.(1996) [13]**	Spain	PCR	Very high	Upper-middle	111	26	4
**Clarridge, J. E., 3rd.et.al.(1996) [10]**	Germany	Immunological detection	Very high	High	46	15	5
**Del AC.et.al(1997) [14]**	China	PCR	High	Low	83	1	4
**Cotte, L.et.al.(1999) [12]**	France	PCR	Very high	High	1454	338	5
**Brasil, P.et.al.(2000) [7]**	Nigeria	Microscopic examination	Low	Upper-middle	40	11	3
**Muller, A.et.al.(2001) [53]**	NA	PCR	NA	High	148	7	3
**Lebbad, M.et.al.(2001) [36]**	Australia	PCR	Very high	Low	52	3	4
**Ferreira, F. M.et.al.(2001) [18]**	Portugal	Immunological detection	Very high	High	860	69	5
**Tumwine, J. K.et.al.(2002) [86]**	Spain	PCR	Very high	Upper-middle	47	8	4
**Lores, B.et.al. (2002) [45]**	Uganda	PCR	Low	Upper-middle	2446	422	5
**Alfa CO.et.al.(2002) [3]**	Yemen	PCR	Low	Low	61	8	4
**Sulaiman, I. M.et.al.(2003) [81]**	Peru	Microscopic examination	High	Upper-middle	2672	105	5
**Abreu-Acosta, Néstor.et.al.(2005) [1]**	Iran	IFAT	High	High	273	25	5
**Espern, A.et.al.(2007) [17]**	Brazil	Microscopic examination	High	Lower-middle	1580	47	5
**Breton, J.et.al.(2007) [8]**	Viet Nam	Microscopic examination	High	High	270	27	5
**Pagornrat, W.et.al.(2009) [58]**	Thailand	IFAT	Very high	Upper-middle	621	75	5
**Tuli, L.et.al.(2010) [85]**	Czechia	PCR	Very high	High	115	22	4
**Sak, B.et.al.(2010) [70]**	India	Microscopic examination	Medium	Low	450	104	5
**Zhang, X.et.al.(2011) [104]**	Czechia	PCR	Very high	High	430	9	5
**Xu N.(2011) [96]**	Czechia	IFA	Very high	Upper-middle	15	7	5
**Sokolova, O. I.et.al.(2011) [80]**	Russia	PCR	Very high	Upper-middle	159	2	4
**Sak, B.et.al.(2011a) [69]**	China	PCR	High	High	40	9	4
**Sak, B.et.al.(2011b) [71]**	China	NA	High	Upper-middle	1996	1	5
**Pavie, J.et.al.(2012) [59]**	Nigeria	PCR	Low	Lower-middle	193	8	4
**Ojuromi, O. T.et.al.(2012) [57]**	France	PCR	Very high	High	143	6	4
**Wang, L.Zhang, H.et.al.(2013) [91]**	Malaysia	PCR	Very high	Lower-middle	447	67	5

**Wang, L.et.al.(2013) [92]**	China	PCR	High	Upper-middle	573	24	5
**Shahrul Anuar, T.et.al.(2013) [76]**	China	PCR	High	Upper-middle	1366	68	5
**Halanova M.et.al.(2013) [26]**	Slovakia	Microscopic examination	Very high	Upper-middle	72	3	4
**Yang, J.et.al.(2014) [99]**	China	PCR	High	Low	252	34	5
**Lobo, M. L.(2014) [44]**	Sao Tome and Principe	PCR	Medium	Upper-middle	348	26	5
**Liu, H.et.al.(2014) [43]**	China	PCR	High	Upper-middle	255	19	5
**Ayinmode, A. B.et.al.(2014) [4]**	Mali	IFA	Low	Lower-middle	132	10	4
**Xie ronghua et.al.(2015) [77]**	Gabon	PCR	High	Upper-middle	200	8	5
**Liu H.(2015) [35]**	India	PCR	Medium	NA	272	16	4
**Hamamc, B.et.al.(2015) [56]**	Türkiye	PCR	Very high	Lower-middle	123	10	4
**Ghoshal, U.et.al.(2015) [46]**	China	NA	High	Upper-middle	252	34	5
**?etinkaya, ü.et.al.(2015) [93]**	China	NA	High	Upper-middle	152	8	4
**Ndzi, E. S.et.al.(2016) [60]**	Cameroon	PCR	Medium	Lower-middle	196	12	4
**Kicia, M.et.al.(2016) [42]**	Guinea-Bissau	PCR	Low	High	86	5	3
**Wang, T.et.al.(2017) [64]**	China	NA	High	Low	285	33	5
**Qiu luyao et.al.(2017) [16]**	Thailand	PCR	Very high	Upper-middle	277	15	5
**Prasertbun, R.et.al.(2017) [106]**	China	PCR	High	Upper-middle	500	1	5
**Liu, H.et.al.(2017) [97]**	China	PCR	High	Upper-middle	124	9	4
**Zhang, Y.et.al.(2018) [25]**	Australia	PCR	Very high	High	605	8	5
**Liu J.(2018) [24]**	Niger	PCR	Low	Lower-middle	93	11	4
**Ding, S.et.al.(2018) [100]**	China	PCR	High	Upper-middle	426	1	5
**Yu, F.et.al.(2019) [90]**	Iran	PCR	High	Lower-middle	102	2	4
**Xu N.(2019) [87]**	Thailand	PCR	Very high	Upper-middle	697	15	5
**Velásquez, J. N., T.et.al.(2019) [61]**	Thailand	PCR	Very high	Lower-middle	200	2	5
**Udonsom, R.et.al.(2019) [48]**	Argentina	PCR	Very high	Upper-middle	143	16	4
**Prasertbun, R.et.al.(2019) [30]**	China	PCR	High	Upper-middle	2284	27	5
**Masoumi-Asl, H..et.al. (2019) [32]**	Iran	NA	High	High	132	10	3
**Halánová, M.et.al.(2019) [79]**	Slovakia	Immunological detection	Very high	High	161	17	4
**Ghoyounchi, R.et.al.(2019) [62]**	China	PCR	High	NA	619	3	5
**Shen, Y.et.al.(2020) [51]**	Mozambique	PCR	Low	NA	1247	9	5
**Muadica, A. S..et.al.(2020) [33]**	China	PCR	High	Upper-middle	609	36	5
**Karimi.et.al.(2020) [22]**	Myanmar	PCR	Medium	Upper-middle	172	15	4
**Ismail, Khadiga Ahmed(2020) [78]**	Poland	PCR	Very high	NA	600	10	5
**Qi, M. et.al.(2020) [72]**	India	NA	Medium	Lower-middle	120	22	3
**Sutthikornchai, C.et.al.(2021) [50]**	France	PCR	Very high	High	456	5	5

**Shehab, A. Y.et.al.(2021) [83]**	south Africa	PCR	High	Upper-middle	170	56	4
**Samie, A.et.al.(2021) [31]**	Egypt	PCR	High	Lower-middle	100	20	4
**Moniot, M.et.al.(2021) [2]**	Thailand	PCR	Very high	Lower-middle	254	4	5
**Kaya, F.et.al.(2021) [54]**	India	NA	Medium	Lower-middle	101	2	3
**Ghoshal, U.et.al.(2021) [69]**	Türkiye	Immunological detection	Very high	Upper-middle	100	4	4
**Naguib, D.et.al.(2022) [71]**	Egypt	PCR	High	Lower-middle	585	27	5
Al-Brhami, K. A. R.et.al.(2022) [91]	Spain	Microscopic examination	Very high	NA	402	57	5
Karim, M. R.et.al.(2022) [92]	Bangladesh	PCR	Medium	Upper-middle	299	19	5
