# Supplementary material for: Global prevalence and risk factors of Enterocytozoon bieneusi infection in humans: a systematic review and meta-analysis
Source: Parasite. 2024 Feb 9;31:9. doi: 10.1051/parasite/2024007 (PMC10860563; doi:10.1051/parasite/2024007)
Supplement: Supplementary file 3 — Table S3: Egger for publication bias. [file parasite-31-9-s3.pdf]

**Table S3.** Egger for publication bias.

| slope | bias | se. bias | t | df | *P*-value |
|---|---|---|---|---|---|
| -1.5031 | -3.5531 | 0.8672 | -4.10 | 73 | 0.0001 |
